# Revision of the endemic Afrotropical genus *Tetractenion* (Hymenoptera, Ichneumonidae) with an identification key to genera of Banchinae for the region

**DOI:** 10.3897/zookeys.1007.55543

**Published:** 2020-12-30

**Authors:** Terry Reynolds Berry, Simon van Noort

**Affiliations:** 1 Research and Exhibitions Department, South African Museum, Iziko Museums of South Africa, P.O. Box 61, Cape Town, 8000, South Africa Iziko Museums of South Africa Cape Town South Africa; 2 Stellenbosch University, Department of Botany and Zoology, Evolutionary Genomics Group, Private Bag X1, Stellenbosch 7602, South Africa Stellenbosch University Stellenbosch South Africa; 3 Department of Biological Sciences, University of Cape Town, Private Bag, Rondebosch, 7701, South Africa University of Cape Town Cape Town South Africa

**Keywords:** Atrophini, Banchini, Glyptini, Ichneumonoidea, Lucid identification keys, taxonomy

## Abstract

The Afrotropical banchine fauna comprises 12 genera: *Apophua* Morley, *Atropha* Kriechbaumer, *Cryptopimpla* Taschenberg, *Exetastes* Gravenhorst, *Glyptopimpla* Morley, *Himertosoma* Schmiedeknecht, *Lissonota* Gravenhorst, *Sjostedtiella* Szépligeti, *Spilopimpla* Cameron, *Syzeuctus* Förster, *Tetractenion* Seyrig, and *Tossinola* Viktorov. A well-illustrated revised key to the genera using high definition images is provided, and the endemic Afrotropical genus *Tetractenion* is revised, previously represented by two described species. Four new species are described: *T.ibayaensis***sp. nov.**, *T.pascali***sp. nov.**, *T.pseudolutea***sp. nov.**, and *T.rosei***sp. nov.** The first species-level identification key is provided for this rare genus. Based on morphological attributes the hypothesis is presented that the species in this genus are probably nocturnal. All images and online interactive Lucid keys are available at: www.waspweb.org and the associated underlying data is made available as Suppl. materials 1, 2 LIF3 files to this paper for inter-exchange with other key production software.

## Introduction

Banchinae is a cosmopolitan group of moderately small to large-sized parasitoid wasps (Gauld et al. 2002). The group is usually well represented in all faunas and amongst the most commonly collected of all ichneumonids (Gauld et al. 2002; Broad et al. 2011). There are roughly 1800 described species and 66 genera of Banchinae currently recognized (Khalaim and Ruíz-Cancino 2012; Watanabe and Maeto 2012, 2014; Broad 2014; Choi et al. 2015; Reynolds Berry and van Noort 2016; Herrera-Florez 2017; Vas 2017; Watanabe 2017, 2018, 2020; Kasparyan and Kulitzky 2018; Li et al. 2018; Sheng et al. 2018; Watanabe and Sheng 2018; Kang et al. 2019, 2020; Yu et al. 2020). With the banchine fauna poorly known for many areas of the world, and a number of undescribed genera and species from tropical regions preserved in museum collections, the number of species is certainly far greater (Broad et al. 2011).

Most Banchinae can be readily diagnosed by the following characters: 1) a submetapleural carina anteriorly generally expanded into a lobe; 2) an arched posterior transverse carina of the propodeum; and 3) a dorsal apical notch on the ovipositor (Wahl and Sharkey 1993; Broad et al. 2011). “However, some or all of these characters do not apply to aberrant genera and species” (Broad et al. 2011) and therefore these morphological characters cannot be classified as synapomorphies for the Banchinae. An additional two apomorphies were later proposed (Gauld and Wahl 2000; Broad et al. 2011): firstly, the subapical flagellomeres of female antennae possess elongate placoid sensilla only on the dorsal surface, with smaller, rounded sensilla on the ventral surface; and secondly, the posterior corner of the pronotum is rounded, slightly twisted and flattened. While within the Ophioniformes group (Ophioninae, Ctenopelmatinae, Banchinae, Mesochorinae, Nesomesochorinae, Metopiinae, Campopleginae, Tatogastrinae, Cremastinae, Tersilochinae, Anomaloninae, Neorhacodinae, Oxytorinae, Stilbopinae, Sisyrostolinae, and Lycorinae; Gauld 1985; Wahl 1991, 1993; Quicke et al. 2009) these two characters are phylogenetically informative, it has been established that they are not synapomorphic for the subfamily Banchinae (Broad et al. 2011).

Morphologically, Banchinae can be subdivided into three tribes namely Banchini, Glyptini, and Atrophini (Townes and Townes 1973; Yu et al. 2020). In addition to having very short ovipositors, Banchini differs from the other tribes by having eight or more sensilla on the larval prelabium (Quicke 2015). Atrophini possess a reduced hypostoma (Quicke 2015) and Glyptini share similar modifications on the metasoma with *Lycorina* and some Pimplinae taxa, in that taxa within Glyptini typically possess triangular areas on tergites II–IV, delimited by paired, lateromedian grooves (Shimizu 2019), though the precise pattern of grooves differs in the former taxa. Given that Pimplinae and Lycorininae are distantly related subfamilies to Banchinae (Bennett et al. 2019), the similarity of these structures is likely to be the result of analogous character states that appear similar as a result of convergence.

Within the Afrotropical region, the subfamily comprises 12 genera and 187 described species: *Apophua* Morley, *Atropha* Kriechbaumer, *Cryptopimpla* Taschenberg, *Exetastes* Gravenhorst, *Glyptopimpla* Morley, *Himertosoma* Schmiedeknecht, *Lissonota* Gravenhorst, *Sjostedtiella* Szépligeti, *Syzeuctus* Förster, *Spilopimpla* Cameron, *Tetractenion* Seyrig, and *Tossinola* Viktorov. A dichotomous identification key to banchine genera within the Afrotropical region was last produced by Townes and Townes (1973), providing the most comprehensive taxonomic treatment to date. Nevertheless, the generic key is outdated and not supported with applicable illustrations of character states. Subsequent to this treatment, the genus *Glyptopimpla* was removed from synonymy with *Teleutaea* Förster and re-instated as a valid genus and as a senior synonym of *Zygoglypta* Momoi and *Orientoglypta* Kuslitzky (Gupta 2002).

*Tetractenion*, placed in the tribe Banchini, is a very rare genus restricted to the Afrotropical region (Townes 1969; Yu et al. 2020). Until now, *Tetractenion* included only two species, *T.luteum* Seyrig, recorded from continental central Africa (Democratic Republic of Congo and Kenya) and *T.acaule* Seyrig, recorded from Madagascar (Seyrig 1932, 1935). The purpose of this paper is to revise the genus *Tetractenion*, and to provide well-illustrated updated keys to the genera of Banchinae in the Afrotropical region, and to the species of *Tetractenion*. Online Lucid keys are available on www.waspweb.org and the associated underlying data is made available as Suppl. materials 1, 2: LIF3 files to this paper for inter-exchange with other key production software.

## Materials and methods

### Photographs

Specimens were either pinned or point mounted on black, acid-free cards for examination (using a Leica M205C stereomicroscope with LED light source), photography, and long-term preservation. Images were taken using the Leica LAS 4.4 system which comprised a Leica Z16 microscope with a Leica DFC450 Camera with a 0.63× video objective attached. The imaging process, using an automated Z-stepper, was managed using the Leica Application Suite V 4.4 software installed on a desktop computer. Diffused lighting was achieved using a Leica Dome. Images of the types held in Musée Royal de l’Afrique Centrale, Tervuren (**RMCA**) were kindly made available by Stéphane Hanot and Arnaud Henrard and those in the Muséum national d’Historie naturelle, Paris (**MNHN**) were kindly made available by Agnièle Touret-Alby. All images presented in this paper are available at www.waspweb.org (van Noort 2020).

### Depositories

Codens follow Arnett et al. (1993), and updated according to the online version http://hbs.bishopmuseum.org/codens/

**NHMUK**The Natural History Museum, London, England (Gavin Broad);

**MNHN** Muséum national d’Historie naturelle, Paris (Agnièle Touret-Alby);

**RMCA** Musée Royal de l’Afrique Centrale, Tervuren (Stéphane Hanot);

**SAMC**Iziko South African Museum, Cape Town, South Africa (Simon van Noort);

**CASC**California Academy of Sciences, San Francisco, United States of America (Robert Zuparko).

### Nomenclature and abbreviations

The morphological terminology follows Wahl and Sharkey (1993), but the wing venation nomenclature follows Gauld (1991). Most morphological terms are also defined on the HymAToL website (http://www.hymatol.org) and HAO website (http://portal.hymao.org/projects/32/public/ontology/). The following morphometric abbreviations are used (in order of appearance in the descriptions):

**A** antenna length, from base of scape to flagellar apex (mm);

**B** body length, from toruli to metasomal apex (mm);

**CT** (clypeus transversality index): maximum width of clypeus: median height;

**F** fore wing length, from tegula to wing apex (mm);

**Fl_n_** (length index of flagellomere n): length: width of flagellomere n;

**IO** (inter-ocellar index): shortest distance between posterior ocelli: ocellus diameter;

**ML** (malar space length index): malar space (shortest distance between mandible base and compound eye): basal mandibular width;

**OO** (oculo-ocellar index): shortest distance between eye and posterior ocellus: ocellus diameter;

**OT** (ovipositor sheath-tibia index): length of ovipositor sheath: length of hind tibia.

The first three measurements (absolute measures) were measured on all specimens in the type series, with measurements from the primary type reported separately in brackets if necessary.

### Identification keys

Identification keys were produced in two formats to facilitate accessibility by a range of end-users and to meet the requirements of publishing both static and dynamic interactive keys under an open access model (Penev et al. 2009; Sharkey et al. 2009): 1. Traditional dichotomous keys these are published below and also made available as interactive keys supported by links to species pages on www.waspweb.org; 2. Online interactive Lucid matrix keys were produced, hosted on www.waspweb.org, and made available as Suppl. materials 1, 2: LIF3 files to this paper for import to, and inter-exchange with other key production software. The LIF3 file is an XML-based file that stores all the Lucid4 key data, allowing exchange of the key with other key developers (Penev et al. 2009; Sharkey et al. 2009). In contrast to dichotomous keys where a choice needs to be made at each key couplet to continue, Lucid matrix keys use a different approach where relevant states from multiple character features can be selected independently until identification is achieved (www.lucidcentral.org). All keys were produced using high quality annotated images, highlighting diagnostic characters that are integrated into the key above each couplet. This is a user-friendly output making the keys readily accessible to a wide range of users with diverse expertise. This key format circumvents the requirement of familiarity with morphological terminology associated with the particular group, because the characters are visually illustrated making the keys usable by the lay person (van Noort et al. 2015).

## Results

### Key to Banchinae genera of the Afrotropical region

**Table:** 

	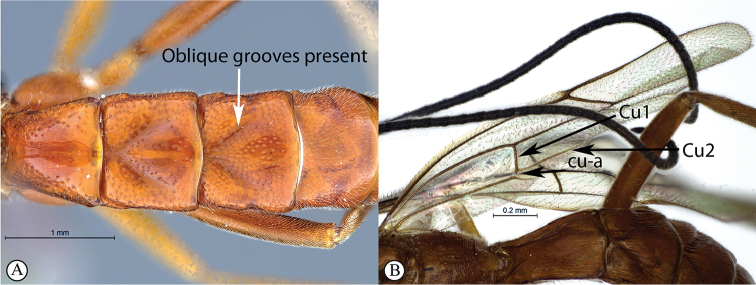	
1	Tergites II–IV with a median pair of (usually) deep oblique grooves that converge anteriorly and diverge posteriorly (A); Cu1 longer than cu-a, such that Cu2 arises below middle of these combined veins (nervellus of Townes) (B)	**2**
	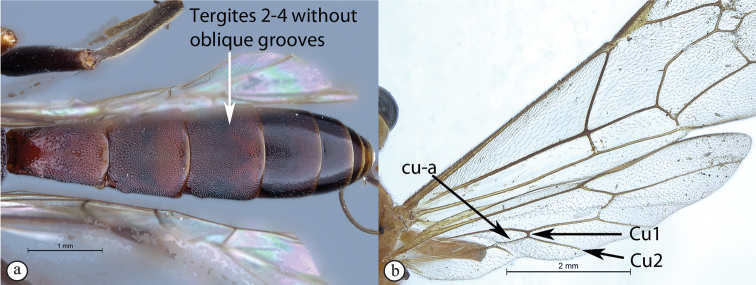	
–	Tergites II–IV without a median pair of oblique grooves (a); Cu1 often longer than cu-a, but may be shorter (b)	**4**
	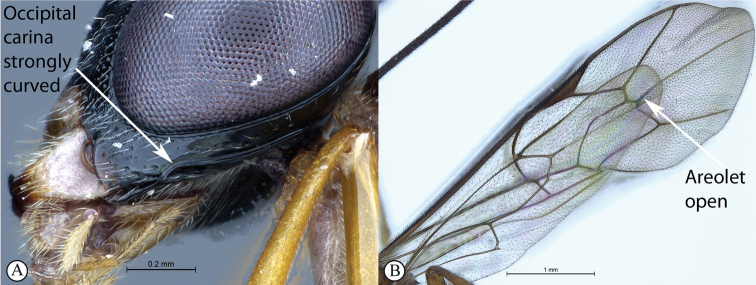	
2	Occipital carina strongly curved before junction with hypostomal carina (A); areolet open, i.e., vein 3rs-m absent (B)	***Apophua***
	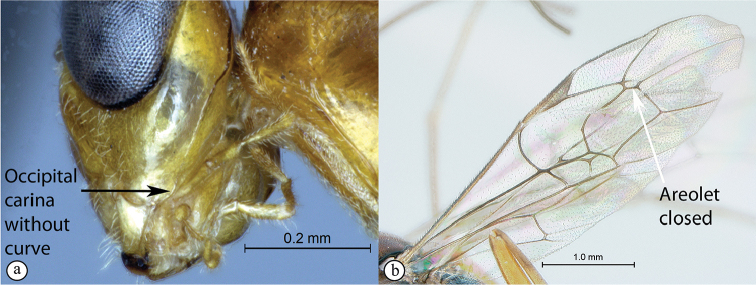	
–	Occipital carina without a strong curve before junction with hypostomal carina (a); areolet closed (b)	**3**
	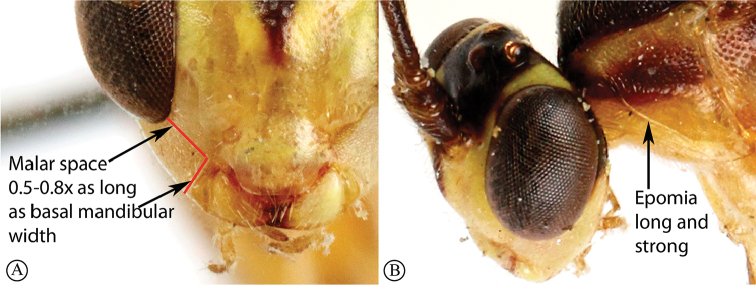	
3	Malar space 0.5–0.8× as long as basal width of mandible (A); epomia long and strong (B)	***Glyptopimpla***
	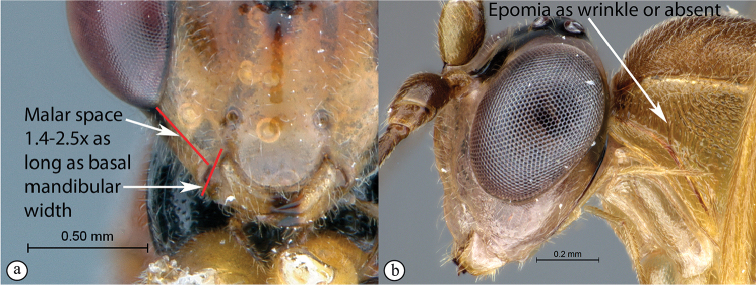	
–	Malar space 1.4–2.5× as long as basal width of mandible (a); epomia usually absent or indistinct, only represented as a short wrinkle (b)	***Sjostedtiella***
	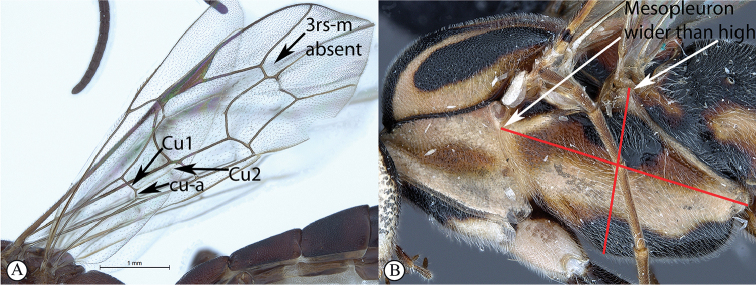	
4	Hind wing with Cu1 longer than cu-a such that Cu2 arises below the middle of these combined veins (nervellus of Townes), Cu2 rarely absent (A); fore wing with 3rs-m sometimes lacking, shape of areolet when closed various (A); mesopleuron usually wider than high (B)	**5**
	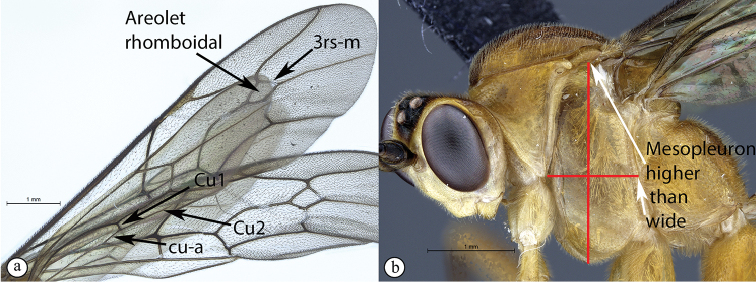	
–	Hind wing with Cu1 shorter than cu-a such that Cu2 arises above the middle of these combined veins (nervellus of Townes) (a); fore wing with 3rs-m always present, areolet rhomboidal (a); mesopleuron usually higher than wide (b)	**11**
	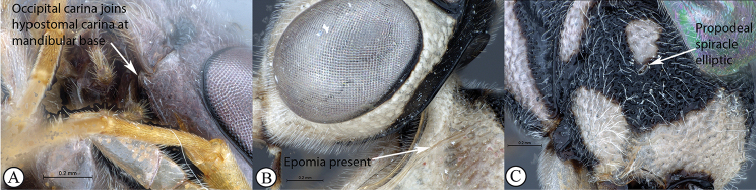	
5	Occipital carina joining hypostomal carina at base of mandible (A); epomia usually present (B); propodeal spiracle elliptic (C)	***Syzeuctus***
	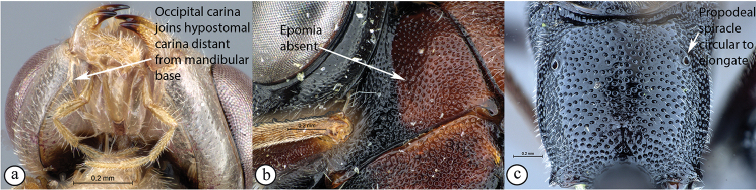	
–	Occipital carina joining hypostomal carina distant from base of mandible (a); epomia usually absent (b); propodeal spiracle circular to elongate (c)	**6**
	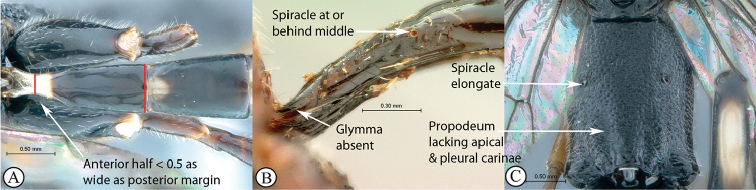	
6	Tergite I with anterior half slender, less than half as wide as posterior margin (A); glymma absent (B); with its spiracle at or behind middle (A, B); propodeum lacking carinae (C); propodeal spiracle elongate (C)	***Atropha***
	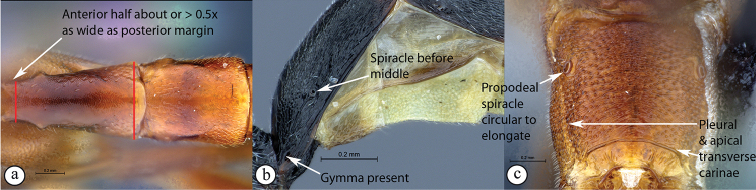	
–	Tergite I with anterior half about or more than half as wide as posterior margin (a); glymma present (a); with its spiracle in front of middle (a, b); propodeum usually with either an apical transverse carina or pleural carina, or both (c); propodeal spiracle usually circular to elongate (c)	**7**
	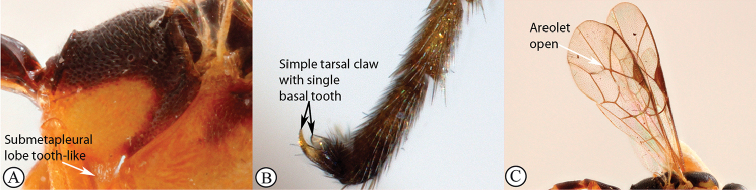	
7	Apex of submetapleural lobe tooth-like (A); tarsal claws simple with a single basal tooth above (B); areolet open (C); occipital carina broadly interrupted above.	***Tossinola***
	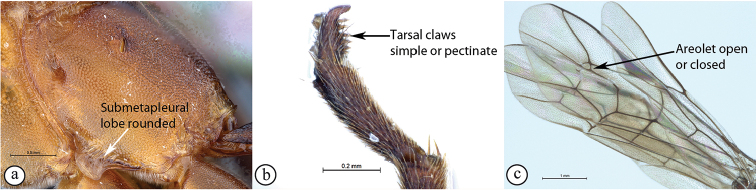	
–	Apex of submetapleural lobe rounded (a); tarsal claws simple or pectinate (b); areolet open or closed (c); occipital carina complete.	**8**
	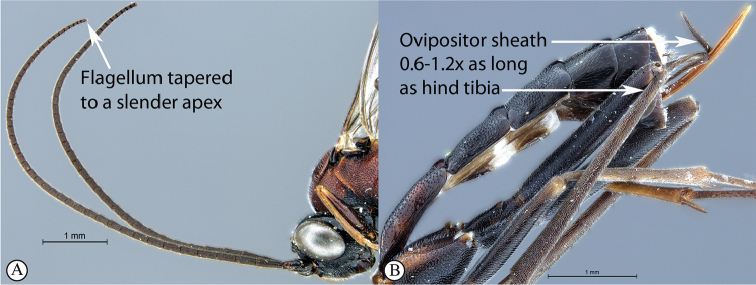	
8	Apical 0.3–0.4 of flagellum tapered to a slender apex (A); ovipositor sheath 0.6–1.2× as long as hind tibia (B)	**9**
	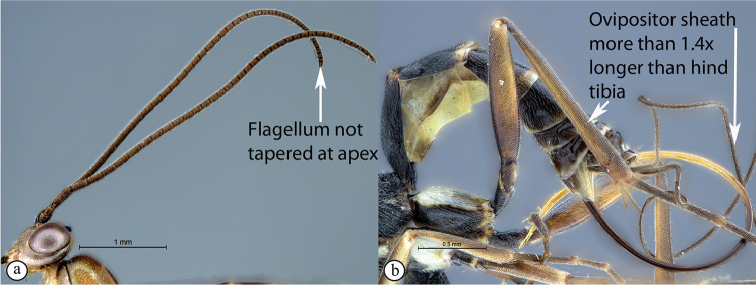	
–	Flagellum not tapered at the apex (a); ovipositor sheath usually more than 1.4× as long as hind tibia (b)	**10**
	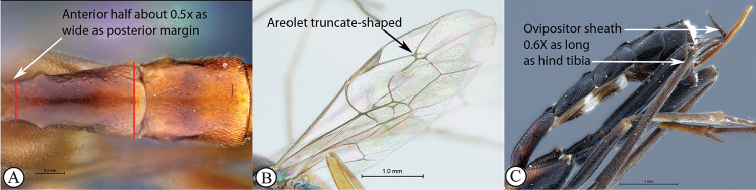	
9	First tergite evenly and rather strongly narrowed anteriorly , about half as wide as posterior margin (A); areolet always truncate-shaped (B); ovipositor 0.6× as long as hind tibia (C)	***Cryptopimpla***
	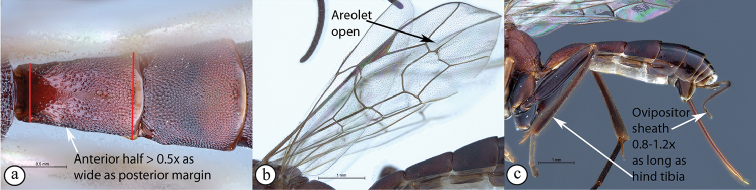	
–	First tergite stout, only moderately narrowed anteriorly, more than half as wide as posterior margin (a); areolet always open (b); ovipositor 0.8–1.2× as long as hind tibia (c).	***Spilopimpla***
	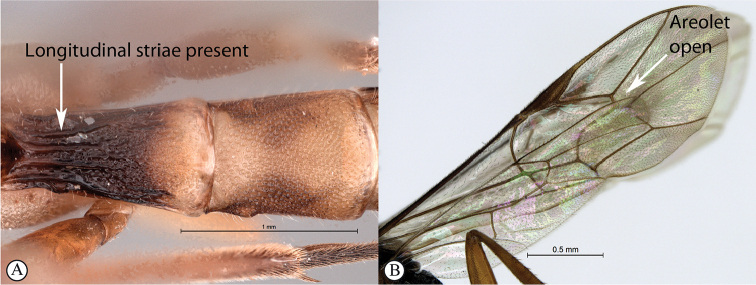	
10	First tergite nearly always with longitudinal striae (A); areolet open (B)	***Himertosoma***
	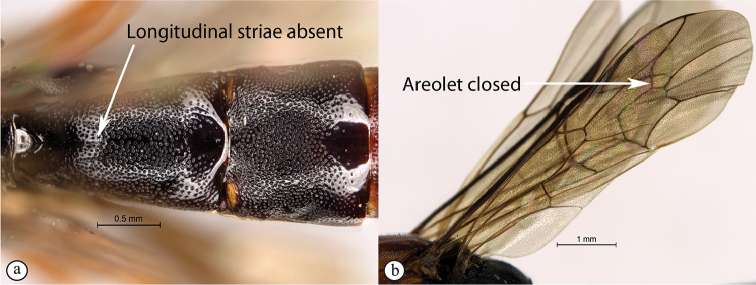	
–	First tergite rarely covered with longitudinal striae (a); areolet closed or sometimes lacking (b)	***Lissonota***
	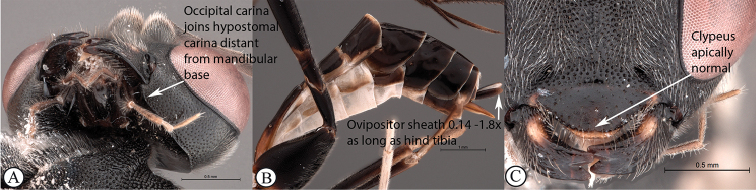	
11	Occipital carina joining hypostomal carina above base of mandible (A); ovipositor sheath short to long, 0.14–1.8× as long as hind tibia (B); mandibular teeth usually subequal in length (C); apical clypeal margin normal (C)	***Exetastes***
	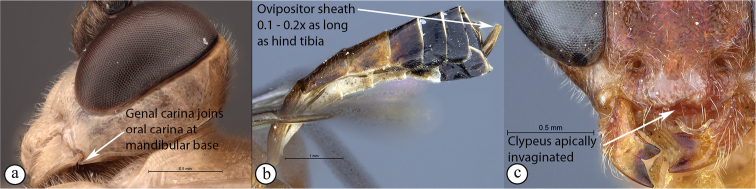	
–	Occipital carina joining hypostomal carina at base of mandible (a); ovipositor sheath always short, 0.1–0.2× as long as hind tibia (b); lower tooth of mandible always longer than upper tooth (c); clypeus apically invaginated (c)	***Tetractenion***

### Details of morphological characters that were used to update the key to Afrotropical banchine genera

1. Occipital carina with strong curve prior to meeting hypostomal carina: this character (couplet 1A), although noted in the global generic key by Townes (1969), was not included in the previously published generic key to Afrotropical Banchinae (Townes and Townes 1973). This is a strong and reliable character distinguishing Apophua from the remaining Glyptini genera, Sjostedtiella and Glyptopimpla.
2. Shape of the areolet when closed: the shape of the areolet has been found to be a useful character to separate the tribes/genera. When distinguishing the tribes Atrophini and Banchini, the areolet in Exetastes and Tetractenion is always large and rhomboidal with a very short stalk, whereas in those Atrophini that possess an areolet it is always small, but variably shaped (couplet 4A, a). An anteriorly truncate areolet (couplet 9B, veins 2rs-m and 3rs-m meeting RS separately) present in many Cryptopimpla species has been reported (Townes 1969; Sheng 2011; Takasuka et al. 2011) to be a character state that is constant for all Afrotropical Cryptopimpla species (Reynolds Berry and van Noort 2016). In Syzeuctus and Atropha, the areolet is triangular with a long stalk and in Lissonota the areolet, when closed, is nearly always petiolate (i.e., dorsal aspect pointed, veins 2rs-m and 3rs-m meet before RS, couplet 10c; Townes 1969).
3. Mesopleuron compressed in Exetastes Group: Tetractenion and Exetastes species have stocky bodies with the mesopleuron laterally compressed (higher than wide) and often flat whereas in Atrophini the mesopleuron is usually wider than high (dorso-ventrally compressed, couplet 4B, b).
4. Distinguishing Tossinola: the length of the ovipositor sheath relative to the hind tibia has previously been used as an additional character to separate Tossinola from the other Afrotropical genera in the tribe Atrophini where the areolet is open (Townes and Townes 1973). However, the relative lengths overlap across Lissonota, Cryptopimpla and Tossinola species, making it an unreliable character to separate these genera. While a medially, broadly interrupted occipital carina is still the most diagnostic character for the genus Tossinola, another useful character is the state of the apex of the submetapleural carinae: tooth-like in Tossinola (Townes 1969) but rounded in the other Afrotropical banchine genera.
5. Flagellum apically tapered: as observed by Townes (1969), the flagella of the genera Lissonota and Himertosoma are not, or may only be weakly, apically tapered. For Cryptopimpla and Spilopimpla species, the flagella are tapered to a slender apex (couplet 8 A, a; Townes 1969; Reynolds Berry and van Noort 2016).
6. Distinguishing Cryptopimpla: аll Afrotropical Cryptopimpla possess a first tergite that is evenly and rather strongly narrowed toward the base (couplet 9A; Reynolds Berry and van Noort 2016). In addition, the ovipositor sheath ca. 0.6× as long as the hind tibia is diagnostic of Cryptopimpla species (couplet 9C; Reynolds Berry and van Noort 2016).
7. Distinguishing Himertosoma from Lissonota: the absence of a crease separating the fifth laterotergite from the fifth metasomal tergite has been suggested as the single defining character that separates Himertosoma from Lissonota (couplet 10A, a; Watanabe and Maeto 2012). However, this does not appear to be a defining character for Afrotropical Lissonota species as the absence/presence of the crease varies within and among species. Given that the areolet can also sometimes be open in Lissonota species, assessment of the sculpture of the first metasomal tergite is required to separate Lissonota from Himertosoma. Himertosoma species nearly always have longitudinal striae present whereas Lissonota species rarely possess either strong punctures or longitudinal striae (couplet 10B, b; Townes 1969).
8. Length of the mandibular teeth: in the global description of the genus Exetastes by Townes (1969), he noted that the length of the lower mandibular tooth relative to the upper could be either equal or slightly longer/shorter. “Slightly” is a poor character description, especially concerning mandibular teeth, which wear out throughout the wasp’s life. In the description of the genus, based on Costa Rican species, by Gauld et al. (2002), all species had equal mandibular teeth. Relative length of the mandibular teeth is a more reliable character, if one of the teeth is markedly longer or shorter. For example, in Afrotropical Cryptopimpla the upper tooth is distinctly longer than the lower tooth (Reynolds Berry and van Noort 2016). Similarly, it has been previously noted (Townes 1969), and further corroborated during this revision of Tetractenion, that the upper mandibular tooth is distinctly shorter in all species, making it a diagnostic feature for the genus. While most Afrotropical Exetastes have equal mandibular teeth, Exetastes discretus (Morley 1917) and an undescribed species in SAMC has mandibles with the lower tooth distinctly longer than the upper. This warrants further investigation, because these two genera are closely related, as they both form part of the Exetastes group. This character may represent a transition between the two genera.
9. Clypeus apically invaginated: this is a diagnostic feature of Tetractenion, while in Afrotropical Exetastes, as has been observed in other species, the clypeal edge is convex or straight, without a median indentation (Gauld et al. 2002).

#### Tetractenion

Taxon classificationAnimaliaHymenopteraIchneumonidae

Seyrig, 1932

936C2604-C3E2-5A6E-A4FE-76721892F599

Tetractenion Seyrig, 1932, Mém. Acad. Malgache 11: 167. Type: Tetractenionacaule Seyrig. Monobasic.

##### Diagnosis

(updated from Townes 1969). Fore wing 6.4–10 mm long. Body of moderate proportions, the hind legs long. Frons unarmed. Head with three lobes on the face, tentorial pits deep; clypeus small, laterally convex with declivity, apically invaginated, with clypeal edge convex. Antennae long and slender, apically tapered. Teeth of mandible both triangular, the lower tooth longer than the upper tooth. Labium not elongate. Occipital carina joining hypostomal carina at the base of mandible. Epicnemial carina present and ending at anterior edge of mesopleuron. Apex of scutellum rounded, notauli present. Propodeum weakly convex, often with transverse wrinkling and with a posterior transverse carina and lateral longitudinal carinae present, but faint or reduced. Pro-and meso-tarsal claws pectinate to apex, meta-tarsal claws pectinate or simple. Areolet is often large and rhomboidal with a short stalk, receiving 2m-cu at center. Fore wing with cu-a opposite 1A or a little distad, ramellus present or absent on 1m-cu. Hind wing with Cu1 shorter than cu-a such that Cu2 arises above the middle of these combined veins. Metasomal tergite I without dorsolateral carinae. Epipleura of tergites II and III ca. 0.15× as wide as long. Posterior third of metasoma moderately laterally compressed. Ovipositor sheath ca. 0.1–0.2× as long as hind tibia.

##### Biology.

Unknown.

##### Distribution.

Angola, Cameroon, Democratic Republic of Congo, Kenya, Madagascar, Namibia, Nigeria, South Africa.

### Species richness

*T.acaule* Seyrig, 1932

*T.ibayaensis* sp. nov.

*T.luteum* Seyrig, 1932

*T.pascali* sp. nov.

*T.pseudolutea* sp. nov.

*T.rosei* sp. nov.

### Key to Afrotropical species of the genus *Tetractenion*

**Table:** 

	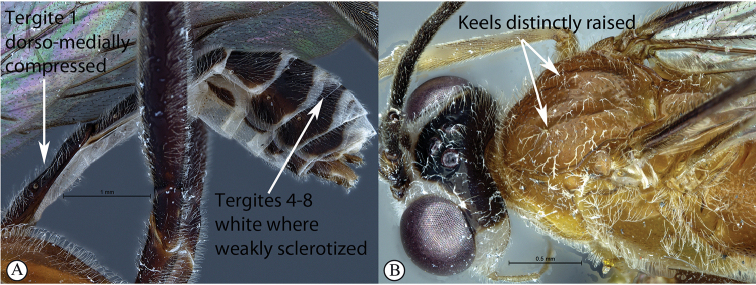	
1	Metasomal tergite I distinctly dorso-medially compressed, tergites IV–VIII white where weakly sclerotized (A); keels distinctly raised on mesoscutal lobes (B); notauli abbreviated, not reaching the scutellum	***T.acaule***
	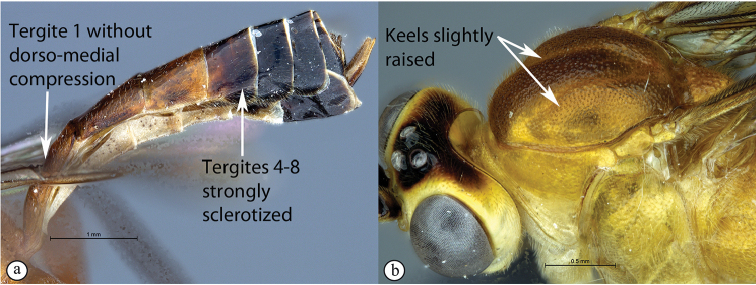	
–	Metasomal tergite I with dorso-medial compression weak or absent, tergites IV–VIII strongly sclerotized (a); keels only slightly raised on mesoscutal lobes (b); notauli present, posteriorly meeting before reaching the scutellum	**2**
	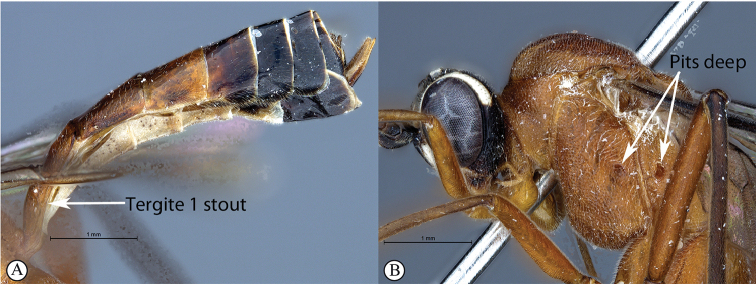	
2	Metasomal tergite I stout, ca. as long as wide in dorsal view (A); pits on the mesopleuron and propodeum are large and deep (B)	***T.ibayaensis* sp. nov.**
	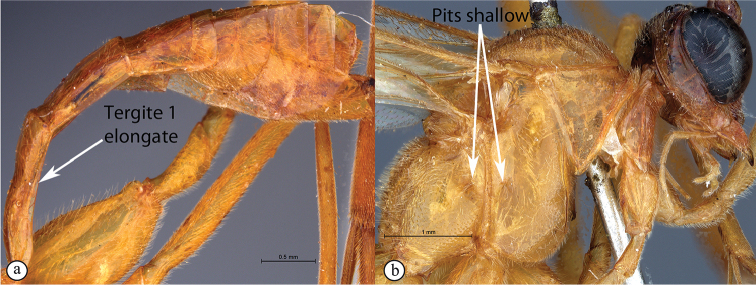	
–	Metasomal tergite I elongate, ca. 2× as long as wide in dorsal view (a); pits on the mesopleuron and propodeum shallow (b)	**3**
	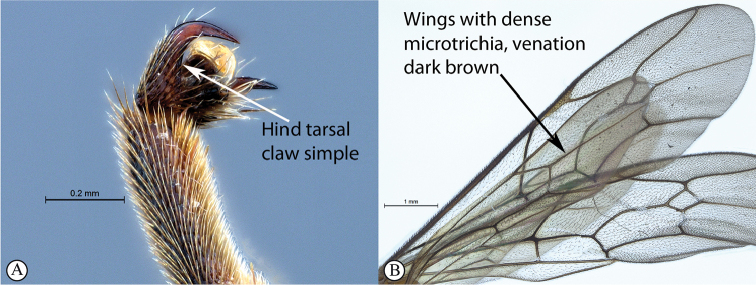	
3	Hind tarsal claw simple (A); wings with dense microtrichia, venation dark (B)	***T.luteum***
	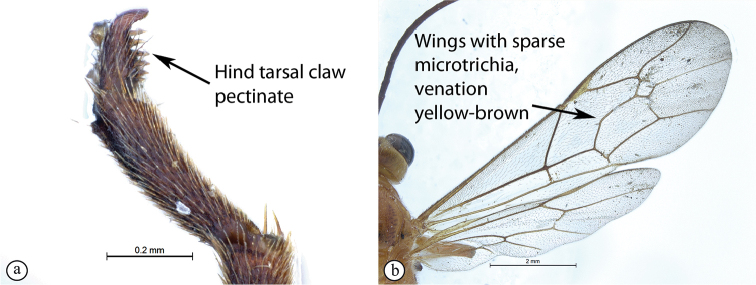	
–	Hind tarsal claw pectinate (a); wings usually with sparser microtrichia, venation usually yellowish-brown (b)	**4**
	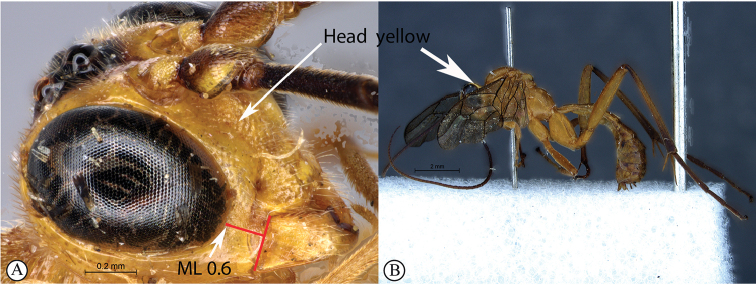	
4	ML 0.6 (A); head yellow, congruent with yellow body (B)	***T.pseudolutea* sp. nov.**
	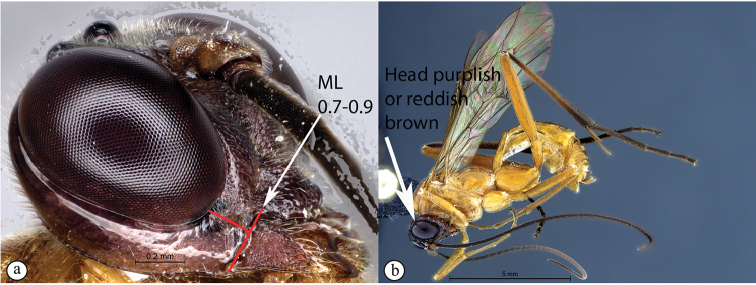	
–	ML 0.7–0.9 (a); head dark purplish-brown or reddish, contrasting with yellow body (b)	**5**
	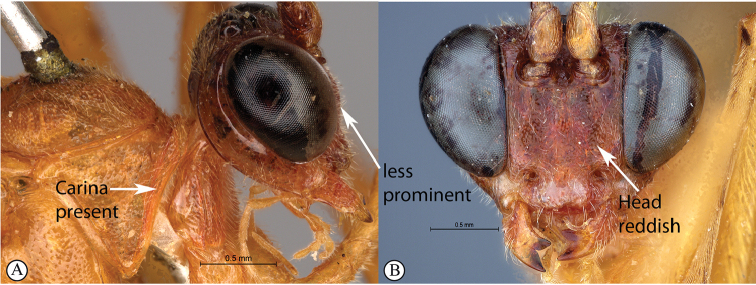	
5	Pronotal collar with strong carina present (A); head reddish and less robust, face weakly three lobed (B)	***T.rosei* sp. nov.**
	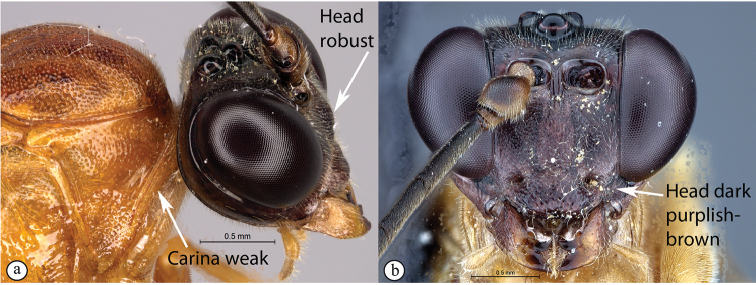	
–	Pronotal collar weakly wrinkled (a); head dark purplish-brown and more robust, face strongly three-lobed	***T.pascali* sp. nov.**

### Species descriptions

#### Tetractenion acaule

Taxon classificationAnimaliaHymenopteraIchneumonidae

Seyrig, 1932

C9C92FC1-C345-57A2-B7A5-1C7FA3FB8613

Fig. 1

##### Type material.

***Lectotype*** ♀: Madagascar, Rogez, Forêt Cote Est, Muséum Paris, 1.31. A. Seyrig, EY9333, [White label with TYPE written in red] [Red type label]: Lectotype ♀ *Tectractenionacuale*, Seyrig, 1932, designated by Townes and Townes (1973), labeled by T. Yoshida, 2011 (MNHN) (photos of Lectotype examined: http://coldb.mnhn.fr/catalognumber/mnhn/ey/ey9333). ***Non-type*** ♀ (examined): Madagascar, Bekily, Reg. Sud. de L’ile, Feb 1930 and Jan–Feb 1931, Coll. Mus. Congo, Col. P.L.G. Benoit, Tectractenionacuale, det. P.L.G. Benoit, 1953 (RMNH). ***Additional material*.** ♀: Madagascar: Majunga Prov., Besalampy District, Marofototra dry forest, 17 km W of Besalampy, 4–11 February 2008, 16°43.30'S, 44°25.42'E, coll. M. Irwin, R. Harin’Hala, Malaise, dry wash in forest, elev. 170 ft MG-42A-20 (CASC).

##### Differential diagnosis.

*Tetractenionacaule* is immediately distinguishable from all other *Tetractenion* species by its unique color combination of a red mesosoma and a mostly black metasoma; distinct keels are present on outer mesoscutal lobes, the notauli do not reach the scutellum; metasomal tergite I has a distinct medial compression in the dorso-ventral view, tergite II have distinct gastrocoeli, and tergites IV–VIII are dorso-posteriorly weakly sclerotized, appearing as large membranous white areas on the dorsal surface. *Tetractenionacaule* closely resembles *T.ibayaensis* as both species are similar in color, having largely fulvous bodies with a white face and the hind femur infuscate, whereas the remaining *Tetractenion* species are largely yellow in color with yellow hind femora. *Tetractenionacaule* can easily be distinguished from *T.ibayaensis* by having a white gena and weakly sclerotized metasomal tergites IV–VIII; the head is narrow, straight behind the eyes; a distinct carina is present on the pronotal collar; distinct keels are present on the outer mesoscutal lobes, with the notauli not reaching the scutellum; pits on the mesopleuron and propodeum are shallow; metasomal tergite I is distinctly dorso-medially compressed; gastrocoeli on tergite II are distinct; tergites IV–VIII are postero-dorsally weakly sclerotized and white; and tarsal claws on the hind leg are simple. In *T.ibayaensis* the gena is brown and tergites IV–VIII are strongly sclerotized; the head is rounded behind the eyes; with no more than a wrinkle present on the pronotal collar; the mesoscutal lobes are hardly present, the notauli reach the scutellum; pits on the mesopleuron and propodeum are deep; metasomal tergite I is stout and indistinctly dorso-ventrally compressed in the medial region; gastrocoeli on tergite II are indistinct; and tarsal claws on the hind legs are pectinate.

##### Description

(updated from Seyrig 1932). Size 9–11 mm. ***Color***: head white with a large black central area on occiput, reaching eyes on vertex and pointed on frons; antenna black, without pale ring; mesosoma red; metasomal tergites I and II red, though tergite II sometimes brownish, following tergites black with large membranous white areas from tergite IV; legs red, hind femur, tibia and tarsus infuscate; wings with sparse microtrichia, venation brown, pterostigma brown and centrally translucent reddish.

***Head*** narrow, straight behind eyes; occiput deeply and angularly excavated, occipital carina strong, extending to lower gena at base of mandible; eyes very large; malar space almost half as long as mandibular basal width; face and clypeus finely, evenly and rather sparsely punctate on a shiny background; face with three lobes, tentorial pits deep; clypeus small, laterally convex with declivity, apically invaginated, with clypeal edge convex; mandibular teeth triangular, lower tooth longer than upper tooth; antenna long, slender and apically tapered.

***Mesosoma*** stout; mesonotum deeply punctate, inter-punctuate spaces about as wide as punctures, rather matt, but not coriaceous; keels distinctly raised on outer mesoscutal lobes of mesoscutum, notauli abbreviated, not reaching the scutellum; apex of scutellum rounded; pronotum shining with a distinct thickened carina on collar, sparsely and very finely punctate; mesopleuron higher than wide, sparsely but more deeply punctate, speculum similarly punctate, background hardly shining, epicnemial carina ending at anterior edge of mesopleuron; shallow pits on mesopleuron and propodeum; metapleuron matt and deeply punctate; propodeum weakly convex, roughly punctate dorsally, punctate posteriorly confluently grading into transverse wrinkles, posterior transverse carina reduced, lateral longitudinal carinae present but faint, spiracle roundish-elliptic and small.

***Metasoma*** hardly punctate at base of tergite II, indistinctly punctate beyond base; tergite I elongate, more than twice as long as wide, tapered anteriorly, glymma present, spiracle positioned slightly in front of middle and protruding, especially dorsally, with a distinct medial depression dorso-ventrally; tergite II longer than wide or subquadrate with gastrocoeli distinct; tergite III quadrate to transverse; metasomal tergites IV–VIII moderately laterally compressed; ovipositor sheath concealed or hardly protruding.

***Fore wing*** without ramellus on Rs-M vein; Rs hardly sinuate; areolet large and quadrate with a short stalk receiving 2m-cu at center. Hind wing with Cu1 shorter than cu-a such that Cu2 arises above the middle of these combined veins. Legs very long; hind femur reaching beyond metasomal apex; length of tibia III plus tarsus III as long as body; spurs of tibia III longer than half metatarsal length; tarsal claws on hind leg simple.

**Male** hardly different: temples a bit less narrowed behind eyes, metasomal tergite II entirely black.

##### Distribution.

Madagascar.

**Figure 1.**
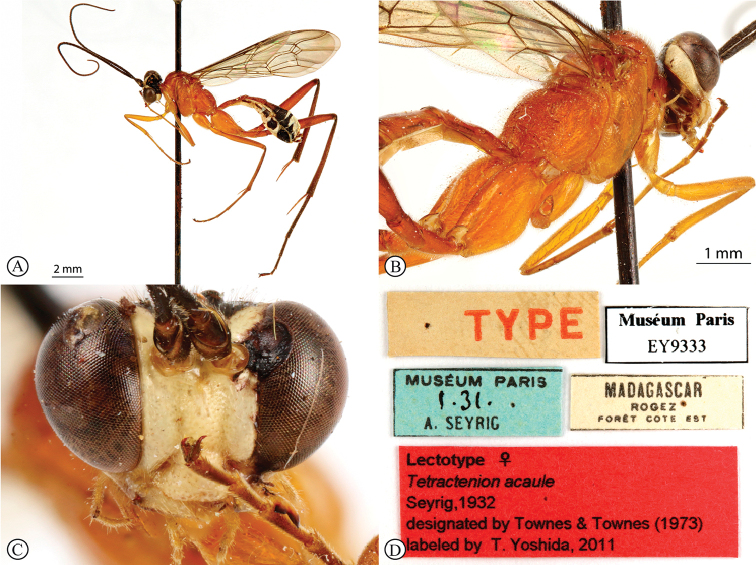
*Tetractenionacaule* Lectotype (MNHN) **A** habitus, lateral view **B** habitus, dorsal view **C** head, anterior view **D** data labels. Photographs of lectotype RECOLNAT (ANR-11-INBS-0004) – Christophe Hervé – 2014. http://coldb.mnhn.fr/catalognumber/mnhn/ey/ey9333 (used with permission of Agnièle Touret-Alby – Curator of HymenopteraMNHN).

#### Tetractenion ibayaensis

Taxon classificationAnimaliaHymenopteraIchneumonidae

Reynolds Berry & van Noort sp. nov.

50E52FFF-BC5C-5BA6-9CCB-FCA596B53778

http://zoobank.org/07849542-A5AB-42C4-80D1-95229B303561

Fig. 2

##### Type material.

***Holotype*** ♀: Tanzania, Mkomazi Game Reserve, Ibaya Camp, north west side, 3°57.91'S, 37°48.09'E, 22–24 April 1996, S. van Noort, *Acacia/Commiphora/Combretum* bushland, Yellow P. Trap, SAM-HYM-P019172 (SAMC).

##### Differential diagnosis.

*Tetractenionibayaensis* is immediately distinguishable from other *Tetractenion* species by having a largely fulvous body and a white face, with the occiput, gena and metasomal tergites IV–VIII dark brown to black, and the hind tibia and tarsus infuscate. The clypeal and mandibular setae are long. The metasoma is hardly laterally compressed with metasomal tergite I stout, being about as long as wide. Pits on the mesopleuron and propodeum are visibly large and deep. In addition, the clypeus is hardly apically invaginated and the propodeal spiracle is distinctly circular and not circular-elliptical as in the other species.

The head is rounded behind the eyes distinguishing the species from *T.acaule* and *T.pascali*. The pronotal collar is no more than a wrinkle, separating the species from *T.acaule* and *T.rosei*. Pectinate tarsal claws on the hind legs separates the species from *T.acaule* and *T.luteum*. Metasomal tergites II and III are quadrate separating the species from all other *Tetractenion* species except *T.acaule* where tergites II and III are sometimes subquadrate and quadrate, respectively; and T.luteum where tergite III is quadrate. Sparse microtrichia on the wings distinguishes *T.ibayaensis* from *T.luteum* and *T.pascali*, and the pterostigma is brown separating the species from *T.luteum*, *T.pascali*, *T.rosei*, and *T.pseudolutea*.

##### Description.

***Body*** mostly fulvous; tibia and tarsus III brown; metasomal tergites IV–VIII brown to nearly black; head with face and area around eyes white; frons and occiput dark brown to near black; mandibles yellow with base and tips brown. Sparse microtrichia on wings, venation and pterostigma brown.

***Head*** rounded behind eyes; occiput deeply and angularly excavated, occipital carina strong, extending to lower gena at mandibular base; malar space half as long as mandibular basal width; eyes very large; face and clypeus finely and evenly punctate on a shiny background; face with three lobes, tentorial pits deep; clypeus small, laterally convex with declivity, apically invaginated, clypeal edge convex; mandibular teeth triangular, lower tooth longer than upper tooth; clypeal and mandibular setae long; antenna long, slender and apically tapered.

***Mesosoma*** stout and deeply punctate on a shiny background; mesopleuron higher than wide, epicnemial carina ending at anterior edge of mesopleuron; deep pits on the mesopleuron and propodeum; pronotum moderately punctate on a shiny background with no more than a wrinkle on collar; mesososcutal lobes hardly present on mesoscutum, notauli posteriorly meeting before reaching the scutellum; propodeum weakly convex, posteriorly confluently grading into weak transverse wrinkles, posterior transverse carina indistinct, lateral longitudinal carinae reduced, spiracle small and circular.

***Metasoma*** with tergite I stout, tapered anteriorly, not distinctly dorso-ventrally compressed in medial region, glymma present, spiracle positioned in front of middle and protruding, especially dorsally, hardly punctate dorso-laterally, metasoma indistinctly punctate beyond and shining; gastrocoeli on tergite II indistinct; tergites II and III quadrate, tergites IV–VIII only slightly higher than wide.

***Fore wing*** without ramellus on Rs-M vein; areolet large and quadrate with a short stalk receiving 2m-cu at center. Hind wing with Cu1 shorter than cu-a such that Cu2 arises above the middle of these combined veins. Legs very long; hind femur reaching beyond metasomal apex, length of tibia III plus tarsus III as long as body; spurs of tibia III longer than half metatarsal length; tarsal claws pectinate.

CT 2.1; ML 0.5; IO 1.6; OO 1.6; Fl_1_ 4.3; OT 0.2; B 8.1 mm; A 8.1 mm; F 6.4 mm.

##### Etymology.

Named after the type locality. Noun in apposition.

##### Distribution.

Tanzania.

**Figure 2.**
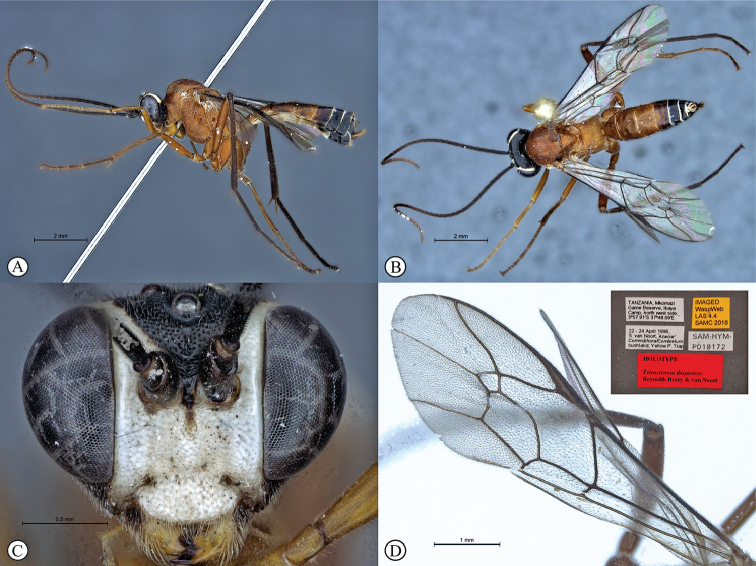
*Tetractenionibayaensis* sp. nov. Holotype **A** habitus, lateral view **B** habitus, dorsal view **C** head, anterior view **D** wing (inset: data labels).

#### Tetractenion luteum

Taxon classificationAnimaliaHymenopteraIchneumonidae

Seyrig, 1935

0A80BD93-D0BD-5639-B0C1-EB219DF96898

Fig. 3

##### Type material.

***Holotype*** ♂: Nyeri, Kenya, June 1932 (MNHN). ***Paratype*** ♀: Elizabethville, Democratic Republic of Congo, 4 January 1921, M. Bequaert, Det. PLG Benoit, 1952 (RMCA). ***Additional material.*** ♀: South Africa, Eastern Cape, Pearston, Plains of Camdeboo Game Reserve, 32°32.033'S, 25°14.267'E, 969 m, 30.x.2009–22.ii.2010, S. van Noort, Malaise Trap, Camdeboo Escarpment Thicket, PCD09-ACA1-M02, SAM-HYM-P047483 (SAMC). ♂, ♀: South Africa, Eastern Cape, Asante Sana Game Reserve, 32°16.762'S, 24°57.309'E, 1186 m, 6.x.2010–17.i.2011, S. van Noort, Malaise Trap, Southern Karoo Riviere Riverine Woodland, ASA09-WOO1-M18, SAM-HYM-P047487 (SAMC, NHMUK). ♂: South Africa, Eastern Cape, Asante Sana Game Reserve, 32°16.762'S, 24°57.309'E, 1186 m, 7 Apr–28 July 2010, S. van Noort, Malaise Trap, Southern Karoo Riviere Riverine Woodland, ASA09-WOO1-M10, SAM-HYM-P047484 (SAMC). ♂: South Africa, Eastern Cape, Asante Sana Game Reserve, 32°15.841'S, 24°57.091'E, 1354 m, 6.x.2010–17.i.2011, S. van Noort, Malaise Trap, Camdeboo Escarpment Thicket, ASA09-BUS1-M17, SAM-HYM-P047485 (SAMC). Namibia, near Windhoek: a bush between kleine Kuppe and Aus Born Mountains, A. Gumovsky, 23–25.xii.2011, SAM-HYM-P047488 (SAMC). ♂: *Exetastes* sp. indet. In B.M. G.J. Kerrich det. 1958. Pres by Com Inst Ent BM 1960-3. U.C. [Nigeria], Ibadan, 9.9.1953, Coll. G.H. Caswell, P49 (NHMUK).

##### Differential diagnosis.

*Tetractenionluteum* is immediately distinguishable from the other species in the genus as this species is the only yellow-colored *Tetractenion* species to possess simple hind tarsal claws, and this character is consistent in both sexes. The head is rounded behind the eyes, distinguishing the species from *T.acaule* and *T.pascali*. The malar space nearly as long as the width of the base of the mandible separates *T.luteum* from *T.acaule*, *T.pseudolutea*, and *T.ibayaensis*. The pronotal collar is weakly wrinkled, separating the species from *T.acaule* and *T.rosei*. Metasomal tergite II is longer than wide and distinguishes the species from *T.ibayaensis*; and a quadrate tergite III separates *T.luteum* from *T.pseudolutea*, *T.pascali*, and *T.rosei*. Furthermore, *T.pascali* is the only other species that possess dense microtrichia on the wings.

##### Description

(updated from Seyrig 1935). Size 7.6–10.4 mm. ***Color***: head yellow with black marking on occiput to middle of frons, no contact with eyes on vertex; meso- and metasoma, fore and mid legs uniformly yellow, hind leg mostly yellow with shades of infuscation on tibia and tarsus infuscate; wings with dense microtrichia, venation brown, pterostigma yellow.

***Head*** with temple short, rounded behind eyes; occiput deeply and angularly excavated, occipital carina strong, extending to lower gena at base of mandible; eyes very large, malar space a bit shorter than width of mandibular base; face and clypeus finely, evenly and rather sparsely punctate on a matt background; face with three lobes, tentorial pits deep; clypeus small, laterally convex with declivity, apically invaginated, with clypeus edge convex; mandibular teeth triangular, lower tooth longer than upper tooth; antenna about as long as body, slender and apically tapered.

***Mesosoma*** stout, matt to sub-polished; pronotum finely punctate on a sub-polished background, no more than a wrinkle present on pronotal collar; mesoscutum moderately punctate, mesoscutal lobes hardly present, notauli posteriorly meeting before reaching the scutellum; mesonotum and mesopleuron finely punctate; mesopleuron higher than wide, epicnemial carina ending at anterior edge of mesopleuron; shallow pits on mesopleuron and propodeum; propodeum weakly convex, matt to sub-polished, moderately punctate posteriorly confluently grading into transverse wrinkles, posterior transverse carina reduced, lateral longitudinal carinae present but faint, spiracle small and circular-elliptical.

***Metasoma*** with a sub-polished background, anterior half of tergite I and dorso-lateral region of tergite II hardly punctate, indistinctly punctate beyond base; tergite I twice as long as wide, glymma present, tapered anteriorly, weak to indistinctly dorso-ventrally depressed in the medial region, spiracle positioned in front of middle and protruding, especially dorsally; tergite II longer than wide, gastrocoeli indistinct; tergite III quadrate.

***Fore wing*** with ramellus absent on Rs-M vein; areolet large and quadrate with a short stalk receiving 2m-cu at center. Hind wing with Cu1 shorter than cu-a such that Cu2 arises above the middle of these combined veins. Legs very long; hind femur reaching beyond metasomal apex, length of tibia III plus tarsus III as long as body, spurs of tibia III longer than half metatarsal length; fore and mid tarsal claws pectinate, hind tarsal claws simple.

##### Distribution.

Democratic Republic of Congo, Kenya, Namibia, Nigeria, and South Africa.

**Figure 3.**
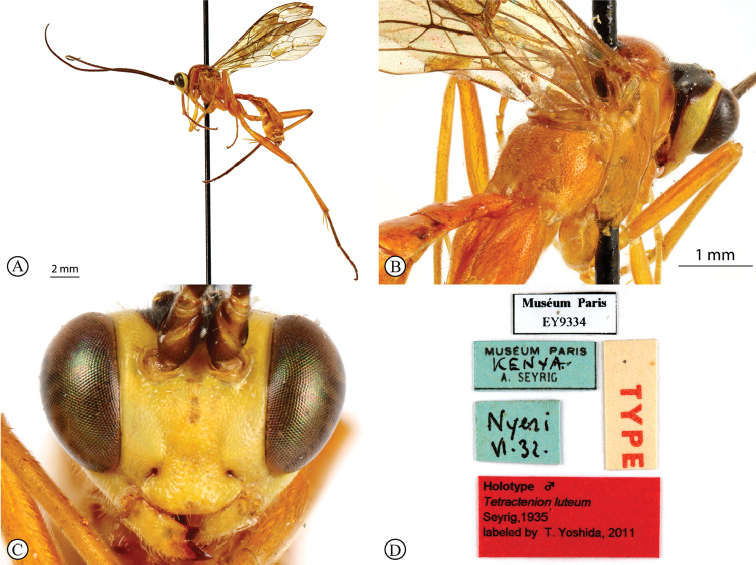
*Tetractenionluteum* Holotype (MNHN) **A** habitus, lateral view **B** habitus, dorsal view **C** head, anterior view **D** data labels. Photographs of holotype RECOLNAT (ANR-11-INBS-0004) – Christophe Hervé – 2014. http://coldb.mnhn.fr/catalognumber/mnhn/ey/ey9334 (used with permission of Agnièle Touret-Alby – Curator of HymenopteraMNHN).

#### Tetractenion pascali

Taxon classificationAnimaliaHymenopteraIchneumonidae

Reynolds Berry & van Noort sp. nov.

C1BDA558-14E7-5FED-9753-3D531AAF9434

http://zoobank.org/026D51D5-49D1-485A-BF2C-784C6ED5F5F0

Fig. 4

##### Type material.

***Holotype*** ♀: Namibia, near Windhoek, between Mandume Ndemufayo Avenue and Western Bypass, 23.xii.2011, SAM-HYM-P047471 (SAMC). ***Paratypes*** ♂: South Africa, Eastern Cape, Asante Sana Game Reserve, 32°16.762'S, 24°57.309'E, 1186 m, 23 Feb–7 April 2010, S. van Noort, Malaise Trap, Southern Karoo Riviere, Riverine Woodland, ASA09-WOO1-M06, SAM-HYM-P044553 (SAMC). ♀: Namibia, near Windhoek: a bush between kleine Kuppe and Aus Born Mountains, A. Gumovsky, 23–25.xii.2011 (NHMUK).

##### Differential diagnosis.

*Tetractenionpascali* is immediately distinguishable from all other *Tetractenion* species by having a color combination of a largely yellow body and a dark head. The facial features are more robust compared to the other species, with the three lobes on the face prominent and the mandibles larger, and the spiracle on the second tergite of the metasoma is hardly protruding. In addition, though the posterior transverse carina may be reduced or faint in the other species, it is distinct in *T.pascali*. The malar space nearly as long as the width of the mandibular base separates *T.pascali* from *T.acaule*, *T.pseudolutea*, and *T.ibayaensis*. Pectinate hind tarsal claws distinguish *T.pascali* from *T.luteum* and *T.acaule*; and a weakly wrinkled pronotal collar separates the species from *T.acaule* and *T.rosei*. Metasomal tergites II and III are longer than wide separating the species from *T.ibayaensis* and *T.acaule*, *T.luteum*, and *T.ibayaensis*, respectively. *Tetractenionluteum* is the only other species besides *T.pascali* that possess dense microtrichia on the wings.

##### Description.

***Color***: head brown, mandibles yellow from base to brown at apex. Antennae brown. Body yellow with red-brown areas on metanotum; tibia III with shades of infuscation, tarsus III infuscate. Wings with dense microtrichia, pterostigma yellow, venation brown.

***Head*** narrowed straight behind eyes; occiput deeply and angularly excavated, occipital carina strong, extending to lower gena at mandibular base; malar space nearly as long as basal mandibular width; eyes very large; face and clypeus features robust, mandibles large; face three-lobed and punctate on a shiny background, punctures on second lobe and clypeus deeper than punctures on lobes flanking eyes, tentorial pits deep; clypeus small, laterally convex with declivity, apically invaginated, clypeal edge convex; mandibular teeth triangular, lower tooth longer than upper tooth; antenna long, slender and apically tapered.

***Mesosoma*** stout and moderately punctate on a shiny background; pronotum with no more than a wrinkle on collar; mesoscutal lobes present on mesoscutum, notauli posteriorly meeting before reaching the scutellum; mesopleuron higher than wide, epicnemial carina present at ending at anterior edge of mesopleuron; shallow pits on mesopleuron and propodeum. Propodeum weakly convex, punctate and posteriorly confluently grading into transverse wrinkles, posterior transverse carina present and distinct, lateral longitudinal carinae present but faint, spiracle small and circular-elliptical.

***Metasoma*** indistinctly punctate on a shiny background; tergite I elongate, twice as long as wide, tapered anteriorly, dorso-ventrally compressed in the medial region, glymma present, spiracle positioned in front of middle and hardly protruding; tergite II longer than wide, gastrocoeli indistinct; tergite III longer than wide; tergites IV–VIII higher than wide.

***Fore wing*** without ramellus on Rs-M vein; areolet large and quadrate with a short stalk receiving 2m-cu at center. Hind wing with Cu1 shorter than cu-a such that Cu2 arises above the middle of these combined veins. Legs very long, hind femur reaching beyond metasomal apex, length of tibia III plus tarsus III as long as body; spurs of tibia III longer than half metatarsal length; tarsal claws pectinate.

**Males**: similar to females; ramellus present.

CT 2–2.4; ML 0.7–0.9; IO 1.2–1.3; OO 1.6–2.1; Fl_1_ 4.5–4.8; OT 0.2; B 7.7–11.5 mm; A 11–14 mm; F 9.2–10 mm.

##### Etymology.

Named after our colleague, Pascal Rousse, who first noted this to be a new species.

##### Distribution.

Namibia and South Africa.

##### Comments.

In males, the ramellus on the fore wing is present, distinguishing the species from *T.acaule* and *T.luteum*. The wings of *T.rosei* are inter-locked; this character could not be compared.

**Figure 4.**
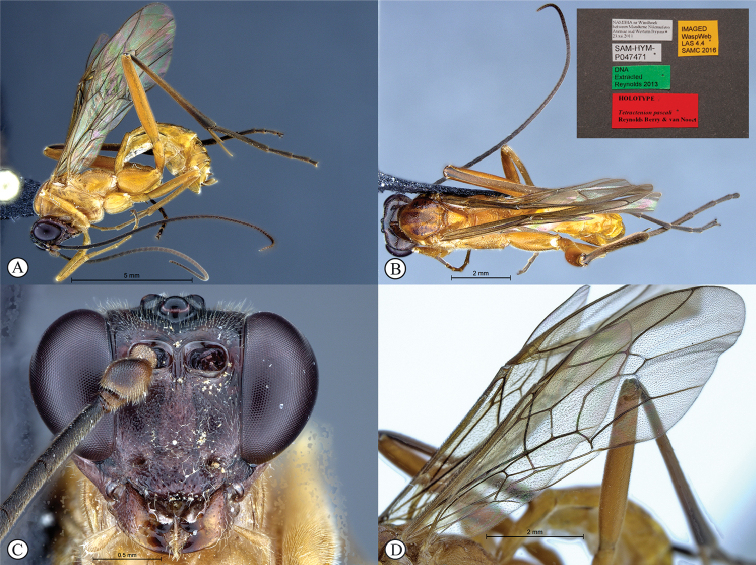
*Tetractenionpascali* sp. nov. Holotype **A** habitus, lateral view **B** habitus, dorsal view (inset: data labels) **C** head, anterior view **D** wings.

#### Tetractenion pseudolutea

Taxon classificationAnimaliaHymenopteraIchneumonidae

Reynolds Berry & van Noort sp. nov.

FA2D41A4-1859-53A7-85F2-AFEAECBC8353

http://zoobank.org/687360CC-2EEA-4136-9B8A-E202F638CC25

Fig. 5

##### Type material.

***Holotype*** ♀: Angola (A11), Bruco, 26.ii–2.iii.1972, Southern African Exp. B.M. 1972-1 (NHMUK). ***Paratypes*** 2♀: Angola (A11), Bruco, 26.ii–2.iii.1972, Southern African Exp. B.M. 1972-1 (NHMUK). ♀: Umbilo, Durban, Natal, 26.10.19, A.L. Bevis, Imp. Inst. Ent. Brit. Mus., 1933-190 (NHMUK). ♂: Cameroon, Ahal, 28.ix.1953. C.I.E. Coll. 15098. Pres. by Com. Inst. Ent. B.M. 1962-1. *Exetastes* sp. det. J.F. Perkins (NHMUK). ♀: Namibia, near Windhoek, between Mandume Ndemufayo Avenue and Western Bypass, 23.xii.2011 [collector not named], SAM-HYM-P047486 (SAMC).

##### Differential diagnosis.

While the color pattern of *Tetractenionpseudolutea* is identical to *T.luteum*, it is distinguishable from *T.luteum* by having pectinate tarsal claw on the hind leg. The head is rounded behind the eyes, separating the species from *T.acaule* and *T.pascali*. The pronotal collar with no more than a wrinkle present distinguishes the species from *T.acaule* and *T.rosei*. Pectinate tarsal claws on the hind leg separates *T.pseudolutea* from *T.acaule* and *T.luteum*. Metasomal tergites II and III are longer than wide distinguishing *T.pseudolutea* from *T.ibayaensis*; and *T.acaule*, *T.luteum*, and *T.ibayaensis*, respectively. Sparse microtrichia on the wings distinguishes the species from *T.luteum* and *T.pascali*; yellowish-brown venation separates the species from *T.acaule*, *T.luteum*, *T.ibayaensis* and *T.pascali*; and a yellow pterostigma distinguishes the species from *T.acaule* and *T.ibayaensis*.

##### Description.

The ***body color*** is the same as in *Tetractenionluteum*, except for density of microtrichia on the wings. *Tetractenionpseudolutea* has sparse microtrichia on the wings with yellow-brown venation, and the pterostigma is yellow.

***Head*** is rounded behind eyes; occiput deeply and angularly excavated, occipital carina strong, extending to lower gena at base of mandible; eyes very large, malar space more than half as long as wide as base of mandible; face and clypeus finely and evenly punctate, background hardly shining; face with three lobes, tentorial pits deep; clypeus small, laterally convex with declivity, apically invaginated, clypeal edge convex; mandibular teeth triangular, lower tooth longer than upper tooth; antennae long, slender and apically tapered.

***Mesosoma*** stout; mesoscutum deeply punctate, mesoscutal lobes hardly present, notauli posteriorly meeting before reaching the scutellum; pronotum finely punctate on a shiny background, no more than a wrinkle present on collar; mesopleuron and mesonotum finely punctate; mesopleuron higher than wide, epicnemial carina ending at anterior edge of mesopleuron; pits on mesopleuron and propodeum are shallow; propodeum weakly convex, finely punctate, posteriorly confluently grading into transverse wrinkles, posterior transverse carina reduced, lateral longitudinal carinae present but faint, spiracle small and circular-elliptical.

***Metasoma*** indistinctly punctate on a shiny background; tergite I twice as long as wide, tapered anteriorly, sometimes weakly dorso-ventrally depressed in the medial region, glymma present, spiracle positioned in front of middle and protruding, especially dorsally; tergite II longer than wide, gastrocoeli indistinct; tergite III longer than wide; tergites IV–VIII moderately laterally compressed.

***Fore wing*** with ramellus rarely present on Rs-M vein; areolet large, quadrate, with a short stalk receiving 2m-cu at the center. Hind wing with Cu1 shorter than cu-a such that Cu2 arises above the middle of these combined veins. Legs very long, hind femur reaching beyond metasomal apex, length of tibia III plus tarsus III as long as body, spurs of tibia III longer than half metatarsal length; tarsal claws pectinate.

CT 2.3; ML 0.6; IO 0.9–1.0; OO 1.7; Fl_1_ 4.6–5.6; OT 0.1; B 9.1–10.7 mm; A 11.3–11.8 mm; F 8.6–9.8 mm.

##### Etymology.

This species at first glance appears to be identical in coloration to *T.luteum* but has morphological differences.

##### Distribution.

Angola, Cameroon, Namibia, and South Africa.

**Figure 5.**
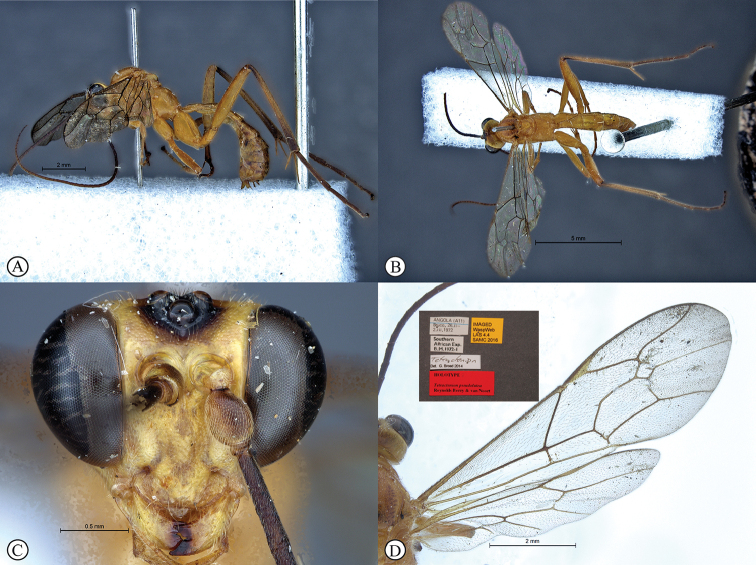
*Tetractenionpseudolutea* sp. nov. Holotype **A** habitus, lateral view **B** habitus, dorsal view **C** head, anterior view **D** wings (inset: data labels).

#### Tetractenion rosei

Taxon classificationAnimaliaHymenopteraIchneumonidae

Reynolds Berry & van Noort sp. nov.

61925215-0094-5E63-A78F-936A6B4EA3CF

http://zoobank.org/A83D4196-5E8B-4D2D-AB29-7F0BDF15C377

Fig. 6

##### Type material.

***Holotype*** ♂: Cameroon, Yaoundé, 1953, C.I.E. Coll. 15098. Pres. by Com. Inst. Ent., B. M. 1962-1. *Exetastes* sp. ♀ det. J. F. Perkins (NHMUK).

##### Differential diagnosis.

*Tetractenionrosei* is immediately distinguishable from other *Tetractenion* species by the reddish color of the head and pronotum in combination with a yellow body, completely yellow legs with venation on the wings also yellow. The head is not narrowed straight behind the eyes but rather rounded, distinguishing the species from *T.acaule* and *T.pascali*. The malar space nearly as long as the basal mandible width separates *T.rosei* from *T.acaule*, *T.pseudolutea*, and *T.ibayaensis*. *Tetractenionacaule* is the only other species besides *T.rosei* possessing a thickened and well-defined carina on the pronotal collar.

Pectinate hind tarsal claws separate the species from *T.acaule* and *T.luteum*. Sparse microtrichia on the wings distinguishes the species from *T.luteum* and *T.pascali*, and the pterostigma is yellow distinguishing the species from *T.acaule* and *T.ibayaensis*. Metasomal tergites II and III are longer than wide separating *T.rosei* from *T.ibayaensis*; and *T.acaule*, *T.luteum*, and *T.ibayaensis*, respectively.

##### Description.

***Color***: head and pronotum reddish, black area restricted to region of ocelli. Body, legs, antennae yellow. Wings with sparse microtrichia, venation yellow, pterostigma yellow.

***Head*** rounded behind eyes; occiput deeply and angularly excavated, occipital carina strong, extending to lower gena at mandibular base; malar space nearly as long as basal mandibular width; eyes very large; face and clypeus moderately and evenly punctate on a shiny background; face with three lobes, tentorial pits deep; clypeus small, laterally convex with declivity, apically invaginated, clypeal edge convex; mandibular teeth triangular, lower tooth longer than upper tooth; antenna long, slender and apically tapered.

***Mesosoma*** stout with a shiny background; mesopleuron moderately punctate, epicnemial carina ending at anterior edge of mesopleuron; pits on the mesopleuron and propodeum shallow; mesonotum moderately punctate; pronotum sparsely and finely punctate on a shiny background with a well-defined carina on collar; mesoscutum deeply punctate, mesoscutal lobes hardly present, notauli posteriorly meeting before reaching the scutellum; propodeum weakly convex, deeply punctate posteriorly confluently grading into transverse wrinkles, posterior transverse carina indistinct, lateral longitudinal carinae present, spiracle small and round.

***Metasoma*** indistinctly punctate on a shiny background; tergite I more than twice as long as wide, tapered anteriorly, slight dorso-ventral depression in medial region, glymma present, spiracle in front of middle and protruding; tergites II and III longer than wide; gastrocoeli on tergite II indistinct; tergites IV–VIII higher than wide.

***Hind wing*** with Cu1 shorter than cu-a such that Cu2 arises above the middle of these combined veins. Legs very long, hind femur reaching beyond metasomal apex, length of tibia III plus tarsus III as long as body, spurs of tibia III longer than half metatarsal length; tarsal claws pectinate.

CT 1.6; ML 0.9; IO 1.4; OO 2.2; Fl_1_ 3.5; B 9.3 mm; F 8.6 mm.

##### Etymology.

Named because of the reddish color of the head and pronotal collar. Noun in apposition.

##### Distribution.

Cameroon.

##### Comments.

This is a rare species known only from one female specimen. Sampling in other areas of the Afrotropical region has so far not produced any further specimens. The wings are inter-locked in such a way that a useful diagnostic character of the wings cannot be seen, i.e., whether the ramellus is present or not.

**Figure 6.**
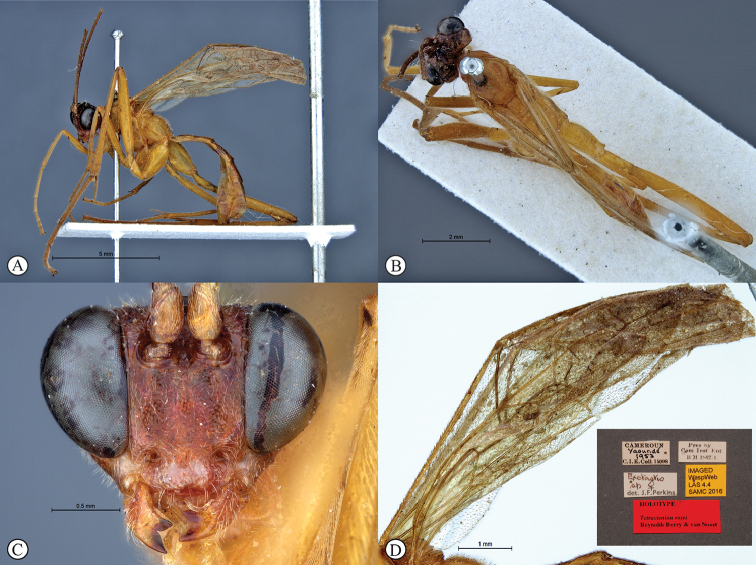
*Tetractenionrosei* sp. nov. Holotype **A** habitus, lateral view **B** habitus, dorsal view **C** head, anterior view **D** wings (inset: data labels).

## Discussion

Since publication of the first key to genera of Banchinae in the Afrotropical region (Townes and Townes 1973), new knowledge on the subfamily has been acquired and there have been recent technological advances allowing for production of good quality images to illustrate relevant diagnostic character states. The revised and updated key is now more user-friendly. The genus *Tetractenion* was previously represented by two species and the present study has yielded an additional four species restricted to the Afrotropical region. Representative specimens in world collections remain rare and apart from those that are housed in the NHMUK and the CASC only; to our knowledge, there are no additional historical specimens present in world collections.

The general habitus and coloration of *Tetractenion* species suggest that this is possibly a nocturnal genus. A list of characters associated with being nocturnal or crepuscular includes a general brown-yellow color; long antennae; large eyes and large ocelli (Gauld and Huddleston 1976; Warrant et al. 2004; Greiner 2006). In addition, most species with this suite of characters are koinobionts (Quicke 2015). The benefit of being a koinobiont nocturnal species is that these parasitoid wasps are able to access hosts that are hidden during the day; many caterpillars conceal themselves during the day and come out to feed at night (Quicke 2015). Diurnal wasps, on the other hand, are faced with the pressures of predation and competition for limited resources (Warrant 2008). Where known, all Banchinae are koinobiont endoparasitoids of Lepidopteran caterpillars (Gauld and Mitchell 1978; Gauld et al. 2002; Fernandes et al. 2010; Broad et al. 2011; Tschopp et al. 2013). *Tetractenion* species possess very large eyes and long antennae relative to body size (A = 8.1–14 mm, B = 7.6–11.5 mm). However, the ocelli were not found to be particularly large (IO = 0.9–1.6, OO = 1.6–2.2); i.e., an ocellus with a large diameter would result in IO and OO indices with values less than one. Like other members of the Banchinae tribe Banchini, *Tetractenion* have very short ovipositors to allow for attack on exposed caterpillars (Fitton 1985, 1987). Members of the tribes Glyptini and Atrophini have ovipositors about as long as or longer than the metasoma and exploit semi- to -concealed hosts in leaf rolls, tunnels, buds, etc. (Townes 1969; Quicke 2015). This provides further support that the genus may have evolved to utilize host resources not readily available during the day. Given the endemicity to the Afrotropical region, *Tetractenion* is also predicted to be a more derived genus within the subfamily Banchinae. Phylogenetic analyses of the subfamily within the Afrotropical region established that the tribe Banchini (only the *Exetastes* group is present in the Afrotropical region), represented by *T.pascali* and two *Exetastes* species, to be the most derived of the three tribes present in the region (Reynolds Berry 2019). Dating of the genus *Tetractenion* could not be determined based on a single species, but given that *Exetastes* has a cosmopolitan distribution and *Tetractenion* is an African endemic it is likely that *Tetractenion* is the more derived of the two genera.

Although there have been recent comprehensive long-term inventory surveys conducted across many parts of Africa with many rich, recently collected bulk samples that still need to be sorted, in reality, comprehensive sampling of Ichneumonidae in the region has been relatively limited to specific areas (Hopkins et al. 2019a, 2019b; Klopfstein et al. 2019; van Noort 2019). The perceived rarity (two species represented by a single specimen) of *Tetractenion* suggests that, with implementation of further rigorous inventory surveys across the many inadequately sampled areas of Africa, there are still more *Tetractenion* species to be discovered. Due to the relatively limited availability of specimens within this genus, any assessments of the distribution and diversification of the different species are still likely to be biased. This is corroborated by new locality records presented in this paper demonstrating bias in previously recorded distributional data: *T.luteum* was previously recorded from the Democratic Republic of Congo and Kenya (Seyrig 1935; Yu et al. 2020), but additional records from southern Africa suggest that this species probably has a more widespread distribution across Africa.

Most of the species have the pro-, meso-, and meta-tarsal claws pectinate to the apex. *Tetractenionluteum* and *T.acaule* (a Madagascan endemic) are the only two species that possess simple tarsal claws on the hind leg. While the overall color patterns of *T.pseudolutea* are identical to *T.luteum*, it is readily distinguishable from *T.luteum* by having a pectinate tarsal claw on the hind leg. With most *Tetractenion* species having pectinate tarsal claws on the hind leg, it is plausible that this character state is the plesiomorphic condition. Based on the assumption that it is more parsimonious for evolutionary trajectories to proceed via the reduction of morphological characters, rather than evolution of more complex character states, *T.luteum* and *T.acaule* are most probably the more derived species within the genus, but this hypothesis requires corroboration with the addition of genetic evidence, and a thorough phylogenetic analyses based on both morphological and molecular characters.

The current revision has increased the species richness of the genus threefold. Further comprehensive sampling will undoubtedly uncover additional *Tetractenion* species in the Afrotropical region.

## Supplementary Material

XML Treatment for Tetractenion

XML Treatment for Tetractenion acaule

XML Treatment for Tetractenion ibayaensis

XML Treatment for Tetractenion luteum

XML Treatment for Tetractenion pascali

XML Treatment for Tetractenion pseudolutea

XML Treatment for Tetractenion rosei

## Supplementary materials

Supplementary material 1 Lucid Interchange Format version 3 (LIF3) for the key to the genera of Afrotropical Banchinae (Hymenoptera, Ichneumonidae) Data type Lucid Interchange Format version 3 Explanation note The LIF3 file is an XML-based file that stores all the Lucid4 key data, allowing exchange of the key with other key developers. https://binary.pensoft.net/file/492596This dataset is made available under the Open Database License (http://opendatacommons.org/licenses/odbl/1.0/). The Open Database License (ODbL) is a license agreement intended to allow users to freely share, modify, and use this Dataset while maintaining this same freedom for others, provided that the original source and author(s) are credited.Terry Reynolds Berry, Simon van Noort

Supplementary material 2 Lucid Interchange Format version 3 (LIF3) and Lucid SDD files for the key to *Tetractenion* species (Hymenoptera, Ichneumonidae, Banchinae) Data type Lucid Interchange Format version 3 (LIF3) and Lucid SDD files Explanation note The LIF3 file is an XML-based file that stores all the Lucid4 key data, allowing exchange of the key with other key developers. https://binary.pensoft.net/file/492597This dataset is made available under the Open Database License (http://opendatacommons.org/licenses/odbl/1.0/). The Open Database License (ODbL) is a license agreement intended to allow users to freely share, modify, and use this Dataset while maintaining this same freedom for others, provided that the original source and author(s) are credited.Terry Reynolds Berry, Simon van Noort
